# Effect of Combined Grape Seed (*Vitis vinifera*) and Garlic (*Allium sativum*) Extracts on Adipogenesis in the 3T3-L1 Preadipocyte Line

**DOI:** 10.3390/ijms27157043

**Published:** 2026-08-06

**Authors:** Silvia García-Dávila, Carmen Cifuentes, Ignacio Gracia, Luís Antonio Gómez, Silvia Llorens

**Affiliations:** 1 Institute of Biomedicine (IB-UCLM), Department of Medical Sciences, Faculty of Medicine of Albacete, University of Castilla-La Mancha (UCLM), 02008 Albacete, Spain; silvia.garciadavila@uclm.es (S.G.-D.); carmen.cifuentes@uclm.es (C.C.); 2 Translational Research Group, GAI of Ciudad Real, 13004 Ciudad Real, Spain; ignacio.gracia@uclm.es (I.G.); luisantonio.gomez@alvenpesalud.com (L.A.G.); 3 Research Institute of Castilla-La Mancha (IDISCAM), 13004 Ciudad Real, Spain; 4 Department of Chemical Engineering, Institute of Chemical and Environmental Technology, University of Castilla-La Mancha (UCLM), 13071 Ciudad Real, Spain

**Keywords:** oligomeric proanthocyanidins condensed, allicin, thiosulfinates, *Allium sativum*, *Vitis vinifera*, adipogenesis, cyclooxygenase

## Abstract

Obesity and its associated metabolic disorders drive the search for natural compounds with anti-adipogenic properties. This study evaluated *Vitis vinifera* seed extract (GE), rich in oligomeric proanthocyanidins (OPCs), and *Allium sativum* bulb extract (ALL), rich in thiosulfinates, for their effects on adipogenesis and COX-related prostanoid modulation in 3T3-L1 cells. Differentiating preadipocytes were treated with non-cytotoxic concentrations of GE, ALL, or their combination (AGE) at a 1:600 dilution (approximately 23 μg/mL allicin and 0.05% OPCs). Lipid accumulation was assessed by Oil Red O staining, prostanoid levels (PGE_2_, TXA_2_, and PGI_2_) were quantified by enzyme immunoassay, and adipogenic gene expression (PPARγ, CEBPα and GLUT4) was analyzed by RT-qPCR. All treatments reduced lipid accumulation, with GE showing the most potent inhibition (68.0% of control), followed by ALL (81.6%) and AGE (78.0%). Distinct prostanoid patterns emerged: GE increased both PGE_2_ and PGI_2_ and decreased TXA_2_, while ALL increased PGE_2_ and reduced both TXA_2_ and PGI_2_. The combination (AGE) did not enhance the anti-adipogenic effects. At the molecular level, GE downregulated PPARγ, CEBPα, and GLUT4, whereas ALL did not affect their transcription, indicating distinct mechanisms. These findings demonstrate that GE and ALL inhibit adipogenesis through differential modulation of the COX pathway and gene expression, highlighting their potential as functional ingredients for obesity management. The lack of synergy in the combination warrants further investigation.

## 1. Introduction

Obesity has reached pandemic proportions worldwide, with its prevalence more than doubling since 1990 and affecting over 890 million adults globally [1]. This complex metabolic disorder represents a major risk factor for numerous non-communicable diseases, including type 2 diabetes, cardiovascular pathologies, and certain cancers, imposing substantial burdens on healthcare systems [2,3,4,5]. The clinical significance of obesity, at the cellular level, depends on the pattern of adipose tissue expansion; hypertrophy (increase in the size of existing adipocytes) and hyperplasia (increase in adipocyte number through the generation of new adipocytes from preadipocytes, a process known as adipogenesis) [6]. Dysregulation of adipogenesis contributes critically to adipose tissue dysfunction, ectopic lipid deposition, and the development of obesity-associated metabolic complications [4,7]. Consequently, understanding the molecular mechanisms governing adipocyte differentiation and identifying natural compounds capable of modulating this process have emerged as promising strategies for obesity management.

Adipogenesis is orchestrated by a complex network of transcription factors, with peroxisome proliferator-activated receptor γ (PPARγ) and CCAAT/enhancer-binding proteins (C/EBPs) serving as master regulators [7]. Beyond transcriptional control, lipid-derived signaling molecules known as prostanoids—synthesized from arachidonic acid via cyclooxygenase (COX) enzymes—have been increasingly recognized as important modulators of adipose tissue biology [8,9]. The prostanoid family includes prostaglandin D_2_ (PGD_2_), prostaglandin E_2_ (PGE_2_), prostaglandin F_2_α (PGF_2_α), prostacyclin (PGI_2_), and thromboxane A_2_ (TXA_2_), each exerting distinct effects through specific G protein-coupled receptors [10]. In the murine 3T3-L1 preadipocyte model—the most extensively characterized system for studying white adipogenesis—PGI_2_ promotes differentiation through IP receptor-mediated signaling and subsequent PPARγ activation [11,12]. Conversely, PGF_2_α potently inhibits adipogenesis via FP receptor activation and downstream calcium–calcineurin pathways [13,14]. The role of PGE_2_ is more complex and context-dependent; while it can exert anti-adipogenic effects through EP3 and EP4 receptors, it has also been shown to promote adipogenesis under certain conditions, reflecting the diversity of its receptor subtypes and downstream signaling [8,9]. Recent investigations have revealed that TXA_2_, traditionally viewed as a pro-thrombotic and pro-inflammatory mediator, negatively regulates the browning process in human adipocytes, antagonizing the pro-adipogenic and pro-thermogenic effects of PGI_2_ [15,16]. Moreover, the TXA_2_-TP receptor axis promotes adipose tissue macrophage M1 polarization and insulin resistance in the context of obesity, further implicating this pathway in metabolic dysregulation [17]. These findings highlight the delicate balance among prostanoid signaling networks in controlling adipocyte fate and function.

Botanical extracts have garnered considerable attention as sources of bioactive compounds with potential anti-obesity properties. Grape (*Vitis vinifera*) extracts, particularly those rich in oligomeric proanthocyanidins condensed (OPCs), exhibit multifaceted biological activities, including antioxidant, anti-inflammatory, and metabolic regulatory effects [18]. Proanthocyanidins have been suggested to interfere with adipogenic pathways, positioning them as candidates for modulating adipose tissue function [19]. Garlic (*Allium sativum*) and its organosulfur compounds, notably allicin and other thiosulfinates, possess well-documented cardioprotective and metabolic benefits [20,21,22,23]. These compounds have been proposed to influence lipid metabolism and adipocyte biology through multiple mechanisms [24,25].

The combination of grape and garlic extracts has previously demonstrated synergistic anti-proliferative effects in colorectal cancer models [26], suggesting that their bioactive constituents may interact cooperatively. However, the potential effects of combined grape and garlic extracts on adipogenesis and their impact on the prostanoid signaling network remain unexplored.

In the present study, we investigated the effects of a standardized grape seed extract (92% OPCs), a standardized garlic extract (Aliben^®^ containing 3.7% allicin and 7% total thiosulfinates), and their combination on adipogenesis in 3T3-L1 preadipocytes. We evaluated cell viability, lipid accumulation, and the production profiles of key prostanoids, PGE_2_, PGI_2_ (measured as its stable metabolite 6-keto PGF_1_α), and TXA_2_ (measured as TXB_2_), to elucidate the potential involvement of the COX-derived signaling pathways in mediating the anti-adipogenic effects of these extracts. Our results demonstrate that *Vitis vinifera* and *Allium sativum* extracts inhibit adipogenesis through distinct prostanoid modulation patterns, with the grape extract showing the most potent effect. Notably, their combination did not enhance anti-adipogenic activity, underscoring the complexity of interactions between bioactive compounds from different natural sources.

## 2. Results

### 2.1. Phytochemical Characterization of the Extracts

To characterize the bioactive compounds present in the extracts used in this study, the phytochemical composition of the grape seed extract (GE), garlic extract (ALL), and their combination (AGE) at a 600-fold dilution was determined by HPLC. As shown in Table 1, the grape seed extract was particularly rich in oligomeric proanthocyanidins (OPCs), with a total OPC content of 902,491 ppm, along with significant amounts of epicatechin (34,091 ppm), proanthocyanidin B2 (23,348 ppm), and epigallocatechin (10,192 ppm). The garlic extract contained allicin (di-allyl thiosulfinate) at 5617 ppm, together with various other thiosulfinates, including allyl-1-propenyl thiosulfinate (31,023 ppm) and dimethyl thiosulfinate (18,302 ppm). After 600-fold dilution, the combination (AGE) yielded final concentrations of 75.2 ppm OPCs and 0.22 ppm allicin, among other compounds. These concentrations correspond to the final concentrations present in the culture medium during the differentiation experiments.

### 2.2. Effect of Extracts on Cell Viability

Prior to evaluating the effects of the extracts on adipogenesis at a single concentration, dose–response experiments were conducted to select the optimal working dilution (see Supplementary Material). 3T3-L1 cells were treated with serial dilutions (1:500 to 1:800) of GE, ALL, and AGE, and both cell viability (MTT) and lipid accumulation (ORO) were assessed. As shown in Supplementary Figures S1 and S2, all extracts inhibited adipogenesis without significant cytotoxicity across the tested range. The 1:600 dilution was selected for subsequent experiments as it represented the lowest concentration that maintained significant anti-adipogenic activity while ensuring maximal safety (viability > 95%).

**Figure 1:**
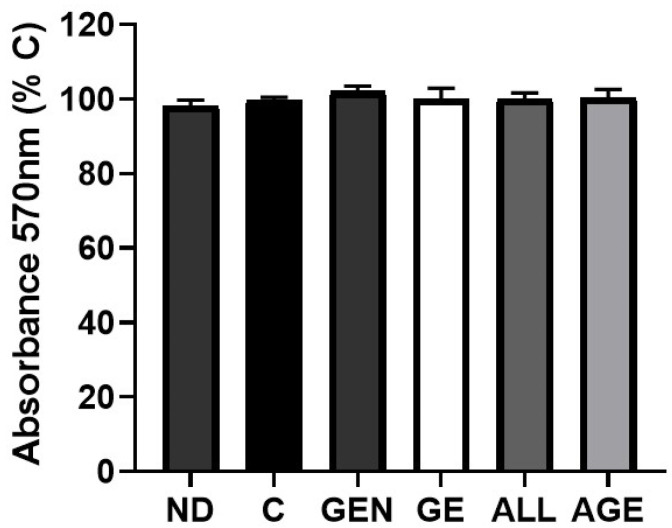
Effect of grape (GE), garlic (ALL) and combined (AGE) extracts on 3T3-L1 cell viability. Differentiating 3T3-L1 preadipocytes were treated with a 1:600 dilution of each extract (GE, ALL and AGE) or genistein treatment (GEN, 12.5 µM). Undifferentiated cells (ND) were incorporated in the assay. Data are presented as mean percentage relative to the differentiation control (C) ± SEM. No statistically significant differences were detected between groups.

To rule out any cytotoxic effects that could interfere with the interpretation of adipogenesis modulation, cell viability was first assessed using the MTT assay in 3T3-L1 cells treated with the selected concentration of each extract (1:600 dilution, corresponding to approximately 23 μg/mL allicin and 0.05% OPCs). As shown in Figure 1, none of the treatments significantly affected cell viability compared to the control (C). Cells treated with *Vitis vinifera* extract (GE), Allium sativum extract (ALL), or their combination (AGE) exhibited viability percentages close to 100% (100.0 ± 13%, 100.1 ± 9.0%, and 100.3 ± 10.9%, respectively), with no statistically significant differences detected. Treatment with genistein (GEN, 12.5 µM) also showed no cytotoxicity compared to the differentiation control (102.2 ± 1.3%). The viability of the undifferentiated cells (ND, 98.1 ± 1.6%) was not altered over the 10-day period compared to the differentiated control cells. These results confirm that the observed effects on adipogenesis and prostanoid production are not attributable to non-specific cytotoxic effects.

### 2.3. Effect of Extracts on Intracellular Lipid Accumulation

**Figure 2:**
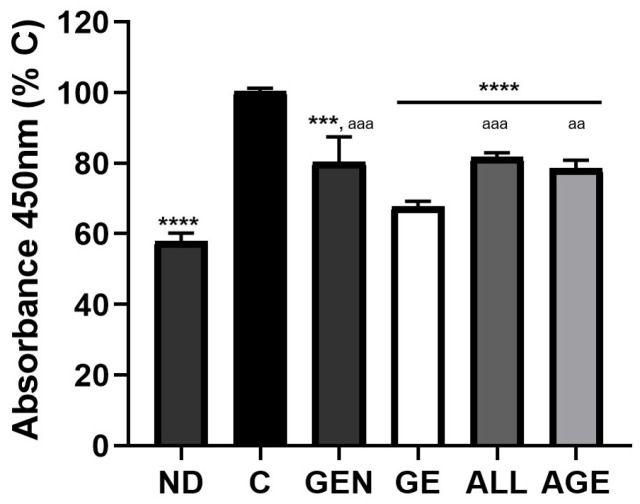
Inhibition of adipogenesis by grape (GE), garlic (ALL) and combined (AGE) extracts. Differentiating 3T3-L1 preadipocytes were treated with a 1:600 dilution of each extract or genistein treatment (GEN, 12.5 µM). Undifferentiated cells (ND) were incorporated in the assay. Data are presented as mean percentage relative to the differentiation control (C) ± SEM. ***, **** *p* < 0.001, *p* < 0.0001 respectively vs. C; aa, aaa *p* < 0.01, *p* < 0.001, respectively vs. GE.

The effect of the extracts on adipocyte differentiation was evaluated by quantifying intracellular lipid accumulation using Oil Red O (ORO) staining. As shown in Figure 2, all treatments significantly reduced lipid accumulation compared to the control (C = 100%). The most pronounced inhibition was observed with GE treatment, which reduced lipid content to 68.0 ± 6.3% of control (*p* < 0.0001). ALL treatment also significantly decreased adipogenesis (81.6 ± 8.8%, *p* < 0.0001), while the combination AGE showed an intermediate effect (78.0 ± 9.8%, *p* < 0.0001). Interestingly, the combination of both extracts (AGE) did not enhance the anti-adipogenic effect compared to GE alone; rather, GE was significantly more effective than AGE (*p* < 0.01) and ALL (*p* < 0.0001). No significant differences were observed between ALL and AGE. As expected, the ND cells produced a significantly lower percentage of lipid content (57.9 ± 0.6%, *p* < 0.0001) compared to the control. In addition, we included a positive control for adipogenesis inhibition by inducing differentiation with genistein (GEN, 12.5 µM) along with the adipogenic cocktail. Genistein is an isoflavone that inhibits adipogenesis [27], so a decrease in lipid content would also be expected. The genistein treatment resulted in a significant 20% reduction in intracellular fat (80.3 ± 1.9%, *p* < 0.0001) compared to the differentiation control. The reduction in intracellular fat produced by GEN was similar to that produced by the ALL or AGE treatments; however, this reduction was statistically less than that produced by the GE treatment (33%) (*p* < 0.0001).

### 2.4. Modulation of COX-Related Prostanoids

To explore the potential involvement of the cyclooxygenase (COX) pathway in the anti-adipogenic effects observed, the levels of three key prostanoids: PGE_2_, TXA_2_ (measured as its stable metabolite TXB2), and PGI_2_ (measured as 6-keto-PGF_1_α), were determined by enzyme immunoassay (EIA).

**Figure 3:**
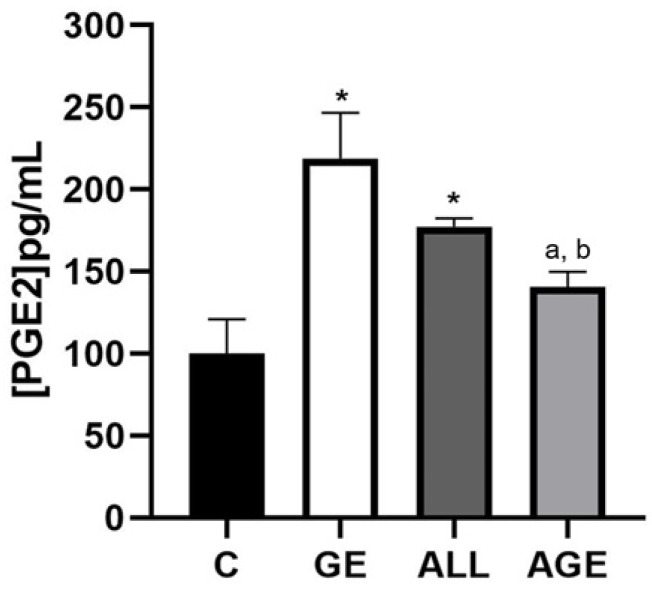
Effect of grape (GE), garlic (ALL), and combined (AGE) extracts on PGE_2_ levels. Differentiating 3T3-L1 preadipocytes were treated with a 1:600 dilution of each extract. Data are presented as mean percentage relative to the differentiation control (C) ± SEM. * *p* < 0.05 vs. C; a *p* < 0.05 vs. GE, b *p* < 0.05 vs. ALL.

For PGE_2_, both GE and ALL treatments significantly increased their levels compared to control (219.0 ± 51.1%, *p* < 0.05 and 177.3 ± 8.7%, *p* < 0.05, respectively). The combination AGE (140.6 ± 18.7%) showed no significant difference from control. A significant difference was observed between ALL, GE and AGE (*p* < 0.05) (Figure 3).

**Figure 4:**
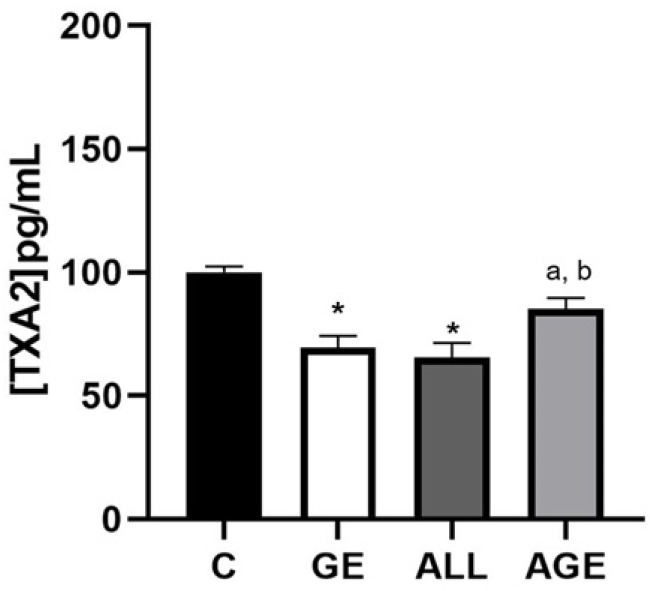
Effect of grape (GE), garlic (ALL) and combined (AGE) extracts on TXA_2_ levels. Differentiating 3T3-L1 preadipocytes were treated with a 1:600 dilution of each extract. Data are presented as mean percentage relative to the differentiation control (C) ± SEM. * *p* < 0.05 vs. C; a *p* < 0.05 vs. GE, b *p* < 0.05 vs. ALL.

Regarding TXA_2_, both GE and ALL significantly reduced its levels compared to control (69.5 ± 11.7%, *p* < 0.05 and 65.4 ± 13.7%, *p* < 0.05, respectively). AGE (85.0 ± 11.2%) did not differ significantly from control. GE and ALL treatments were significantly different from AGE (*p* < 0.05 for both comparisons) (Figure 4).

**Figure 5:**
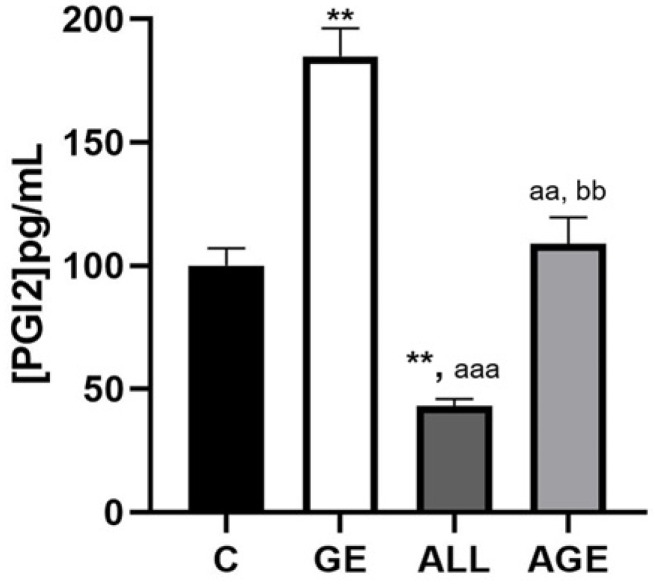
Effect of grape (GE), garlic (ALL) and combined (AGE) extracts on PGI_2_ levels. Differentiating 3T3-L1 preadipocytes were treated with a 1:600 dilution of each extract. Data are presented as mean percentage relative to the differentiation control (C) ± SEM. ** *p* < 0.01 vs. C; aa, aaa *p* < 0.01, *p* < 0.001 respectively vs. GE, bb *p* < 0.01 vs. ALL.

For PGI_2_, a strikingly divergent pattern was observed. GE significantly increased PGI_2_ levels (184.9 ± 22.7%, *p* < 0.05 vs. C), whereas ALL dramatically reduced them (43.1 ± 5.0%, *p* < 0.05 vs. C). AGE (109.0 ± 18.5%) showed no significant difference from control. The differences between GE and ALL (*p* < 0.001) and between GE and AGE (*p* < 0.01) were highly significant (Figure 5).

### 2.5. Effects on Adipogenic Gene Expression

To gain mechanistic insight into the anti-adipogenic effects of the extracts, we analyzed the expression of key adipogenic transcription factors, PPARγ and CEBPα, and GLUT4 by RT-qPCR, where expression is directly regulated by PPARγ and serves as a functional marker of PPARγ activity. Cells were treated with GE and ALL at the selected 1:600 dilution, and undifferentiated cells (ND) and genistein (GEN, 12.5 μM) were included as controls. Data were obtained from at least two independent experiments. Expression levels were normalized to GAPDH and expressed relative to the differentiated control (C).

**Figure 6:**
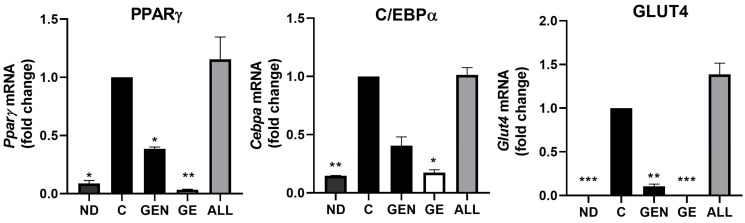
Effects of grape seed extract (GE), garlic extract (ALL), and genistein (GEN) on the expression of adipogenic genes in 3T3-L1 cells. Total RNA was extracted at day 10 post-differentiation, and the expression of PPARγ, CEBPα, and GLUT4 (a downstream marker of PPARγ activity) was analyzed by RT-qPCR. Data represent mean ± SEM from at least two independent experiments. Expression was normalized to GAPDH and expressed relative to the differentiated control (C). * *p* < 0.05; ** *p* < 0.01; *** *p* < 0.001 vs. C.

ND cells exhibited very low expression of all three markers, confirming the validity of the differentiation model. Treatment with GE significantly reduced PPARγ expression by 96.5% (0.035 ± 0.003, *p* < 0.01), CEBPα by 82.7% (0.173 ± 0.025, *p* < 0.05), and GLUT4 by 99.8% (0.0022 ± 0.0007, *p* < 0.001) compared to control (C). This potent transcriptional suppression is consistent with the strong inhibition of lipid accumulation observed with GE (68.0% of control). In contrast, ALL did not significantly alter PPARγ (1.16 ± 0.19), CEBPα (1.01 ± 0.06), or GLUT4 (1.34 ± 0.13) expression compared to control (C). Although a trend toward upregulation was observed for GLUT4 (39%), the difference was not statistically significant. These results indicate that ALL’s anti-adipogenic effect (81.6% of control) is not mediated through transcriptional suppression of adipogenic master regulators. Genistein (GEN), used as a positive control, reduced PPARγ expression by 62% (0.39 ± 0.02, *p* < 0.05), CEBPα by 60% (0.4 ± 0.1), and GLUT4 by 90% (0.10 ± 0.03, *p* < 0.001) compared to control (C). While the reduction in CEBPα did not reach statistical significance, the reductions in PPARγ and GLUT4 were significant, confirming that GEN acts as an inhibitor of adipogenesis at the transcriptional level, albeit less potently than GE. These gene expression results are shown in Figure 6.

## 3. Discussion

The present study demonstrates that both *Vitis vinifera* (rich in oligomeric proanthocyanidins condensed, OPCs) and *Allium sativum* (rich in thiosulfinates) extracts exert significant anti-adipogenic effects in 3T3-L1 cells at non-cytotoxic concentrations. This finding is of interest in the context of adipopathy or obesity, as the regulation of adipogenesis has been proposed as a potential therapeutic target for reducing fat mass [28]. These findings are consistent with previous reports on the bioactivity of these natural extracts in different cellular models [25]. The selected concentration (1:600 dilution, corresponding to approximately 23 μg/mL allicin and 0.05% OPCs) was based on previous studies by our research group demonstrating the bioactivity of these extracts without compromising cell viability [26,29].

The anti-adipogenic effect observed with the grape seed extract is consistent with previous studies demonstrating that proanthocyanidins reduce lipid accumulation in 3T3-L1 adipocytes [30]. Similarly, the inhibitory effect of the garlic extract aligns with reports by Lestari et al. [25] showing that garlic-derived compounds suppress adipogenesis in the same cellular model. These concordant findings validate our experimental model.

The grape seed extract (GE) showed the most potent inhibition of lipid accumulation, significantly outperforming both the garlic extract alone and their combination. This aligns with the well-documented anti-adipogenic properties of proanthocyanidins, particularly OPCs, which have been shown to modulate adipogenesis through multiple mechanisms, including the regulation of adipogenic transcription factors such as PPARγ and C/EBPα [19]. Interestingly, a recent study on encapsulated garlic extract also reported significant inhibition of adipogenesis in 3T3-L1 cells, accompanied by decreased expression of PPARγ, C/EBPα, GLUT4, and adiponectin [25]. Our data extend these observations by demonstrating that, unlike garlic, grape seed extract exerts its anti-adipogenic effect primarily through potent transcriptional suppression of PPARγ and CEBPα, while garlic appears to act through distinct, post-transcriptional mechanisms.

To further elucidate the molecular mechanisms underlying these effects, we analyzed the expression of key adipogenic transcription factors (PPARγ and CEBPα) and the adipocyte maturation marker GLUT4 by RT-qPCR. GLUT4 was included as a downstream marker of PPARγ activity, since its expression is directly regulated by PPARγ and CEBPα [31]. Given that prostanoids such as PGI_2_ and PGE_2_ modulate PPARγ activity through their respective receptors (IP and EP4) [32,33,34], measuring GLUT4 expression allowed us to assess whether the changes in prostanoid levels observed in our study translate into functional changes in the adipogenic transcriptional program.

Our results show that GE significantly reduced PPARγ, CEBPα, and GLUT4 expression, confirming that its anti-adipogenic effect is mediated through transcriptional suppression of the adipogenic program. This is consistent with previous studies showing that grape seed proanthocyanidins modulate the expression of these genes in adipose tissue [35]. In contrast, ALL did not significantly alter PPARγ, CEBPα, or GLUT4 expression, indicating that its anti-adipogenic effect is not mediated through transcriptional suppression. This suggests a post-transcriptional mechanism, such as modulation of protein activity, phosphorylation, or receptor signaling. In fact, Lestari et al. [25] reported decreased protein levels of PPARγ, CEBPα, and GLUT4 in 3T3-L1 cells. However, in that study, the extract was administered on days 8–12 post-differentiation, whereas our ALL extract was applied with the adipogenic cocktail during the first 48 h of differentiation. Further studies are needed to elucidate the precise post-transcriptional pathways involved in the anti-adipogenic effect of ALL.

The lack of a synergistic or additive effect in the combination treatment (AGE) was unexpected. The observation that GE alone was significantly more effective than AGE, while ALL alone and AGE showed comparable effects, suggests that the presence of garlic components may interfere with the anti-adipogenic activity of grape proanthocyanidins. Although direct evidence of antagonism between these compounds is lacking, both allicin and proanthocyanidins have been independently reported to modulate PPARγ expression and activity [30,32], a master regulator of adipogenesis. Specifically, allicin has been shown to reduce the expression of genes involved in lipid synthesis, including PPARγ, both in vitro and in vivo [32], while proanthocyanidins regulate PPARγ during adipogenesis [30]. However, our RT-qPCR results reveal that ALL does not suppress PPARγ or CEBPα at the transcriptional level, suggesting that its reported effects on PPARγ may occur at the protein level rather than through transcriptional regulation. It is plausible that competition for this common molecular target could underlie the observed lack of synergy. This mechanistic divergence could explain why ALL does not enhance, but rather attenuates, the potent transcriptional inhibition exerted by GE, possibly through chemical interactions or by activating pathways that counteract the effects of grape polyphenols. Further studies are warranted to explore potential interactions at the level of receptor binding or intracellular pathway crosstalk.

The analysis of COX-related prostanoids provides valuable insights into the potential mechanisms underlying the anti-adipogenic effects. Prostanoids play complex and sometimes opposing roles in adipogenesis [33,34]. The COX pathway, comprising COX-1 and COX-2, generates prostaglandin H_2_ (PGH_2_), which is subsequently converted by specific synthases into various prostanoids, including PGE_2_, PGD_2_, PGF_2_α, PGI_2_, and TXA_2_ [33]. PGI_2_ is considered a pro-adipogenic prostaglandin because it upregulates *C/EBPβ* and *C/EBPδ* expression by activating the IP receptor in preadipocytes, and it thereby promotes adipogenesis [11]. Furthermore, the activation of IP receptors by PGI_2_ is also suggested to activate PPARγ [36]. On the other hand, PGE2 and PGF2α are considered anti-adipogenic prostaglandins; they suppress adipogenesis by activating their G protein-coupled receptors, EP4 and FP receptors, respectively [13,14,37,38].

Our results reveal distinct modulation patterns for each prostanoid depending on the treatment. PGE_2_, which has been implicated in both pro- and anti-adipogenic effects depending on the context and receptor subtype engagement [33], was significantly increased by GE and ALL treatment. As mentioned above, the EP4 receptor for PGE_2_ has been specifically involved in the suppression of 3T3-L1 adipocyte differentiation [33], suggesting that the increased PGE_2_ observed might contribute to the anti-adipogenic effects through this receptor.

The reduction in TXA_2_ levels by both GE and ALL is particularly noteworthy. TXA_2_ is traditionally associated with platelet aggregation and is consequently a key target for antiplatelet therapy. This reduction by these natural extracts may have potential as a complementary approach to antiplatelet therapy, given that it is known that in obesity the therapeutic effect of antiplatelet treatments is diminished or altered [39,40,41]. Aspirin is less effective at blocking thromboxane synthase in obese patients, and an increase in urinary TXB2 has been observed in obese patients, indicating increased TXA_2_ levels compared with non-obese patients [42]; however, its role in adipogenesis is less understood. The significant decrease observed with both individual extracts suggests a common mechanism, possibly involving downregulation of TXA_2_ synthase or reduced substrate availability. Our findings suggest that these extracts may have potential as complementary agents for further investigation, although in vivo studies are required to validate their efficacy and safety before any therapeutic application can be considered.

The most striking finding was the opposite modulation of PGI_2_ by GE (increase) and ALL (decrease). PGI_2_ (prostacyclin) and its metabolite 15-deoxy-Δ^12^,^14^-PGJ_2_ (15d-PGJ_2_) are known ligands for PPARγ, a master regulator of adipogenesis [33]. The dual role of PPARγ in adipogenesis is well established: while it is essential for adipocyte differentiation, its activation by certain ligands can also modulate the differentiation process [43,44]. The increase in PGI_2_ by GE might reflect enhanced production of prostacyclin, potentially leading to PPARγ-mediated effects, but it contrasts with the reduced intracellular lipid production by GE. We speculate that GE may attenuate the activation of the IP receptor by PGI2, preventing the progression of the early phase of adipogenesis triggered by *C/EBPβ* and *C/EBPδ*, which would otherwise be increased. Conversely, the dramatic reduction in PGI_2_ by ALL could indicate inhibition of PGI_2_ synthase or diversion of the common precursor PGH_2_ toward other prostanoid pathways. Reduced PGI_2_ production by the ALL extract would lead to decreased *C/EBPβ* and *C/EBPδ* expression; thus, the inhibition of adipogenesis by the ALL extract may result not only from the rise in anti-adipogenic PGE_2_, but also from the reduction in PGI_2_ production. While our results demonstrate distinct modulation of prostanoid levels by GE and ALL, the underlying enzymatic mechanisms remain to be elucidated. Future studies examining the expression and activity of prostanoid-related enzymes, including COX-1, COX-2, PGI_2_ synthase, and TXA_2_ synthase, would help to clarify whether the observed changes in prostanoid production are due to transcriptional regulation or direct modulation of enzyme activity.

The integration of prostanoid and gene expression data provides a coherent mechanistic framework. The divergent effects of GE and ALL on PGI2, combined with their similar anti-adipogenic outcomes, suggest that these extracts act through different mechanisms. GE appears to promote a pro-adipogenic prostanoid profile with high PGI_2_ that might favor PPARγ-mediated signaling; however, it also promotes an anti-adipogenic profile by increasing PGE2 and decreasing TXA_2_, while ALL reduces both TXA_2_ and PGI_2_ and increases PGE_2_. Our mRNA expression results support this mechanistic divergence: GE acts primarily at the transcriptional level (suppressing PPARγ and CEBPα), while ALL appears to act through post-transcriptional mechanisms, possibly involving prostanoid receptor signaling (EP4 activation and IP receptor inhibition). This mechanistic divergence could explain why their combination did not result in additive effects, as the opposing modulation of PGI_2_ might lead to functional antagonism. Overall, while our data demonstrate a clear correlation between changes in prostanoid levels and the inhibition of lipid accumulation, further studies are required to establish a causal relationship between these events.

The relationship between prostanoid modulation and adipogenesis inhibition observed in our study is consistent with the complex role of the COX pathway during adipocyte differentiation. Chu et al. [45] reported that stable overexpression of either COX-1 or COX-2 in 3T3-L1 preadipocytes resulted in a marked reduction in triacylglycerol accumulation, suggesting that altered prostanoid biosynthesis can directly impact adipogenesis. Furthermore, the differentiation-dependent regulation of the COX cascade, with significant decreases in prostaglandin release following differentiation [8], supports the notion that prostanoid levels are tightly regulated during adipogenesis and that exogenous compounds modulating this pathway can influence the differentiation process.

Regarding prostanoid involvement, our results extend previous observations that grape seed proanthocyanidins decrease COX-2 expression in both adipose tissue and cultured adipocytes [19], providing a potential mechanistic link between COX-derived signaling and the anti-adipogenic effects of these extracts.

In summary, our integrated phenotypic and molecular data reveal that GE exerts its potent anti-adipogenic effect through transcriptional suppression of PPARγ and CEBPα, while ALL acts through distinct, post-transcriptional mechanisms, possibly involving prostanoid receptor signaling. The lack of synergy between GE and ALL suggests functional antagonism, highlighting the complexity of combining bioactive compounds from different natural sources. These findings provide a foundation for future mechanistic studies aimed at elucidating the specific molecular targets of these extracts in adipocytes.

## 4. Materials and Methods

### 4.1. Preparation and Chemical Characterization of Vitis vinifera and Allium sativum Extracts

The white grape seed polyphenol extract from *Vitis vinifera* utilized in this investigation was prepared following an established protocol. Initially, the grape seeds were milled, and the resulting material underwent a solid–liquid extraction process. This process used water as the solvent, with pH adjustments using sulfuric acid (96%), and continued until the solid phase was depleted of extractable compounds. Following stabilization with sulfites to avoid activating fermentation processes in the raw material, the extract was subsequently dried using a column atomization technique under rigorously controlled atmospheric pressure and temperature below 100 °C to maintain the stability of the polyphenolic compounds. In the atomization column, the water was evaporated with the application of heat, obtaining a solid final product with a concentration of up to 92% in proanthocyanidins. The water activity of the product was measured to be below 0.3 units, according to procedures performed in AVIALSA, located at Ctra. Munera (CM-3119) No. 7, 02600 Villarrobledo, Albacete, Spain, the manufacturer. The remaining presence of sulfites was lower than 500 ppm in the final product rich in proanthocyanidins, according to Spanish legislation for food products.

For garlic, allicin was selected as the reference compound because it is the most biologically reactive—and therefore the least stable—compound, making it a suitable quality marker. For grape seeds, we focused on total proanthocyanidin content (OPCs), as these compounds possess multiple reactive centers and exist as isomeric structures that differ only in spatial arrangement; the aggregate OPC content is more informative for dose–response relationships than the quantification of any single compound. These criteria make it possible to establish a reliable and effective dose–response relationship.

The proanthocyanidin profile of the resulting extract, which is particularly rich in OPCs with a degree of condensation greater than 4 monomeric units, was characterized by HPLC. The chromatographic separation was achieved using a Zorbax Sb-aq column (4.6 × 150 mm, 5 µm particle size, Agilent Technologies, Inc., Santa Clara, CA, USA). The mobile phase consisted of a gradient mixture of water, methanol, acetonitrile, and formic acid (all HPLC grade), following a specific elution program at a controlled temperature of 28 °C. The flow rate was 1 mL/min, the injection volume was set to 50 µL, and the eluted compounds were monitored at a wavelength of 280.4 nm. Quantification was performed using a set of external standards, including determination of gallic acid, (+, −) epicatequin, (+, −) catequin, (+, −) epigalocatequin; as well as oligomeric proanthocyanidins A1, A2, B1, B2, C1, and OPCs, all supplied by Analysis Vínicos (Tomelloso, Spain). The gradient program used is shown in Table 2.

The *Allium sativum* extracts used in this study were Aliben^®^, a commercial product supplied by Alvenpe Salud SL (Plaza de la Reina No. 2, 16660 Las Pedroñeras, Cuenca, Spain). This specific extract is produced from purple garlic from the La Mancha region, employing a methodology protected under Spanish patent number ES2306597 B1. This procedure guarantees a minimum allicin content of 3.7% and a total thiosulfinate content of 7%. To determine the concentration of thiosulfinates in the Aliben^®^ extract, analyses were conducted on a Shimadzu HPLC system (Disburg, Germany). Separation was carried out on a Supelcosil C18 column (150 × 4.6 mm; 5 µm particle size) using an isocratic mobile phase composed of a methanol/water (50:50, *v*/*v*) mixture delivered at a flow rate of 1 mL/min. Compound detection was achieved with a UV-vis detector set to a wavelength of 254 nm. Dimethyl sulfoxide served as the internal standard for these analyses, which was also obtained from Analysis Vínicos (Tomelloso, Spain).

### 4.2. Preparation of Stock Solutions

The stock solutions were prepared as follows: the extracts were dissolved in sterilized phosphate-buffered solution (PBS), centrifuged at 4000 rpm for 15 min, and filtered through a 0.45 µm pore-size filter (Millipore, Bedford, MA, USA). A stock solution of the garlic extract (ALL) was prepared in PBS to a concentration that, upon subsequent dilution, yielded a final allicin concentration of approximately 23 μg/mL in the culture medium. A stock solution of the grape seed extract (GE) was prepared by dissolving 4.1 g of the extract powder in 10 mL of sterile PBS. This stock solution was then diluted 600-fold in differentiation medium. A combination of ALL and GE was also assayed (AGE). The dilution for assay (600-fold) was freshly prepared from the stock solutions in the medium of induction used to differentiate 3T3-L1 adipocytes, to obtain a concentration of 0.5% Proanthocyanidins and 2.3% allicin. The combination ratios of Allicin/OPC’s were 4.6:1. This methodology and ratios of concentration were based on previous studies [26,29]. The cytotoxicity of the extracts at the selected concentrations was previously evaluated in our group in HT 29 and Caco2 cell lines [29]. The 1:600 dilution was selected based on preliminary dose–response experiments (see Supplementary Material S1 and S2 for full details). This dilution was chosen because it maintained cell viability above 95% for all treatments while producing significant inhibition of adipogenesis (Suplementary material, Table S1) and represented the lowest concentration that sustained maximal anti-adipogenic activity (Suplementary material, Tables S2 and S3). At this dilution, the final concentrations in the culture medium were approximately 23 μg/mL allicin (for ALL) and 0.05% OPCs (for GE).

### 4.3. Cell Culture

The 3T3-L1 preadipocyte cell line (American Type Culture Collection, Manassas, VA, USA) was used to test these compounds on adipogenesis. Cells were cultured in Dulbecco’s Modified Eagle’s Medium (DMEM, 90%) and supplemented with 10% inactivated fetal bovine serum (FBS), L-glutamine (1%), and penicillin/streptomycin (0.5%) and maintained at 37 °C in a humidified atmosphere containing 5% CO_2_. Upon reaching 70–80% confluence, cells were counted using an automated counter (TC10 Automated Cell Counter, Bio-Rad, Hercules, CA, USA) via the Trypan Blue exclusion test (0.4% Trypan Blue) [46]. Cells were seeded in 96-well culture plates at a cellular density of 30 × 10^3^ cells/mL. Once optimal confluence was reached, the cells were subjected to the differentiation protocol (as detailed in Section 4.4) in the presence or absence of the extracts for 48 h. Undifferentiated cells (ND), cultured in growth medium without the differentiation cocktail, were included as a baseline control to determine basal levels of lipid accumulation and cell viability. Additionally, genistein (GEN, 12.5 μM), a well-characterized isoflavone known to inhibit adipogenesis [27], was used as a positive control for the suppression of adipocyte differentiation. At the end of the differentiation period (at day 10 post-differentiation), cell viability and intracellular lipid accumulation were assessed (see Section 4.5 and Section 4.6), and the supernatants were collected, centrifuged, and stored at −20 °C until required for subsequent analysis of prostanoids (see Section 4.7). 3T3-L1 cells at passages 6 and 7 were used for all experiments, and three independent experiments were performed for each assay.

### 4.4. Induction of Adipogenesis

Cells were differentiated with induction medium (DMI), consisting of culture medium supplemented with an adipogenic cocktail (0.5 mM IBMX, 0.25 μM dexamethasone, and 1 μg/mL insulin). Forty-eight hours after the initiation of differentiation, DMI was replaced with adipocyte maintenance medium consisting of culture medium supplemented with 1 μg/mL insulin until full differentiation was achieved (approximately 10 days after induction) [47]. Cells were considered mature adipocytes when they exhibited large, intracellular lipid droplets. Extracts were added to the 3T3-L1 cultures simultaneously with the DMI. The concentrations of the extracts were 0.5% proanthocyanidins and 2.3% allicin. The combination ratio of allicin/OPCs was 4.6:1. Individually, the grape seed extract contained a guaranteed 92% OPCs, and the garlic extract a guaranteed 3.7% allicin and a minimum 7% total thiosulfinates. To assess the interaction between GE and ALL, both extracts were also tested individually. Differentiated cells that were not treated served as the differentiation control.

### 4.5. Determination of Cell Viability

Assessment of cell viability was performed using a colorimetric method based on the MTT assay (3-(4,5-dimethylthiazol-2-yl)-2,5-diphenyltetrazolium bromide). This technique relies on the capacity of metabolically active cells to reduce the yellow tetrazolium salt to purple, insoluble formazan crystals via mitochondrial enzymes, thereby providing an indirect measure of the viable cell population. Subsequent solubilization of the formazan precipitate with DMSO yields a violet-colored solution suitable for quantitative analysis [48].

At ten days post-induction of 3T3-L1 cells differentiation, the culture medium was carefully removed, and the cell monolayer was rinsed with DMEM without phenol red and FBS. A freshly prepared solution of MTT in PBS (0.5 mg/mL) was then added to each well at a volume of 100 µL, and the plates were incubated in darkness for 45 min at 37 °C. After this period, the MTT solution was aspirated and replaced with an equivalent volume of DMSO to dissolve the formazan crystals. The plates were subjected to gentle agitation for 3–5 min to ensure complete solubilization. Absorbance measurements were subsequently recorded at a wavelength of 570 nm using a microplate spectrophotometer. (ASYS UVM 340, Cambridge, UK). The maximum absorbance detected for the differentiation control was defined as 100% cell viability, and the viability of the differentiated adipocytes in the presence of extracts, GEN, or ND was expressed as a relative percentage of the control.

### 4.6. Determination of Intracellular Lipid Accumulation

Upon completion of the differentiation protocol, the cultured cells were subjected to Oil Red O (ORO) staining following established methodologies [49,50]. This lipophilic dye exhibits a high affinity for neutral lipids, particularly triacylglycerols, making it a valuable tool for the quantitative assessment of adipogenic differentiation. The ORO stock solution was prepared 24 h prior to the staining procedure by dissolving 0.2 g of the dye in 100 mL of isopropanol. This mixture was maintained under continuous agitation at room temperature in darkness throughout the preparation period. On the day of the experiment, the working solution was freshly prepared by combining the stock solution with double-distilled water in a 6:4 ratio (*v*/*v*). This mixture was subsequently passed through two layers of Whatman filter paper to eliminate any undissolved particles or precipitates. For the staining procedure, the cell monolayer was initially rinsed three times with PBS to remove residual culture medium. Fixation was then performed by exposing the cells to 10% formaldehyde for 15 min at room temperature. Following careful removal of the fixative, the preparations were briefly washed with cold PBS and allowed to dry for 10 min. The freshly filtered ORO working solution was then applied to ensure complete coverage of the cellular surface. After a 10 min incubation period, the dye solution was aspirated, and the cells underwent three additional washes with cold PBS, followed by a 15 min air-drying step. For quantitative analysis, the incorporated ORO stain was extracted using 100% isopropanol. The optical density of the resulting solution was determined spectrophotometrically at a wavelength of 450 nm using a microplate reader (ASYS UVM 340, Cambridge, UK). The measured absorbance values, which correlate directly with the intracellular lipid content, were derived from a minimum of ten replicates per experimental condition, obtained from three independent experiments.

### 4.7. Measurement of COX-Related Prostanoids: TXA_2_, PGI_2_, and PGE_2_

To evaluate the potential involvement of the cyclooxygenase (COX) pathway in the anti-adipogenic effects of the extracts, the levels of three key COX-derived prostanoids—thromboxane A_2_, TXA_2_ (measured as its stable metabolite TXB_2_), prostacyclin I_2_, PGI_2_ (measured as 6-keto-PGF_1_α), and prostaglandin E_2_, PGE_2_—were determined in the culture supernatants by enzyme immunoassay (EIA). For this, we used commercial kits (Cayman Chemical, No. 519031, 515211, and 514531, Ann Arbor, MI, USA), according to the instructions provided by the manufacturer. Briefly, using 96-well plates, standards and samples were incubated with TXB2, 6-keto PGF1α, and PGEM AChE Tracer and EIA Antiserum for 18 h at room temperature for the PGEM and TXB2 EIA kits and at 4 °C for 6-keto PGF1α. The previous step required for the PGEM EIA kit was as follows: the supernatants and PGEM standard were derivatized by adding carbonate buffer and incubated at 37 °C overnight, followed by the addition of phosphate buffer and EIA buffer to a total volume of 1 mL. After incubation with tracer and antiserum, the plates were washed five times, and the Ellman’s reagent was added. The plates were placed on an orbital shaker and developed in the dark for an additional 60 min. The enzymatic reaction in these EIA kits produces a yellow color, the intensity of which is inversely proportional to the amount of TXB2, 6-keto PGF1α, and PGEM present in the wells during the incubation. The absorbance was determined by spectrophotometry at a wavelength of 410 nm using a microplate reader (ASYS UVM 340, Cambridge, UK). Results are reported as pg/mL.

### 4.8. Quantitative Real-Time PCR Analysis: RT-qPCR

Total RNA was extracted from 3T3-L1 cells at day 10 post-differentiation using the PureLink™ RNA Mini Kit (Thermo Fisher Scientific Inc., Waltham, MA, USA) according to the manufacturer’s instructions. RNA quantity and integrity were assessed spectrophotometrically using a NanoDrop™ One (Thermo Fisher Scientific Inc., Waltham, MA, USA). Complementary DNA (cDNA) was synthesized from 1 μg of total RNA using the RevertAid H Minus First Strand cDNA Synthesis Kit (Thermo Fisher Scientific Inc., Waltham, MA, USA). The reaction was carried out in a C1000 Touch Thermal Cycler (Bio-Rad) following the manufacturer’s protocol. The reaction was carried out in a thermocycler (Bio-Rad) under the following conditions: 72 °C for 5 min, 42 °C for 60 min, and 70 °C for 10 min. Gene expression was assessed by quantitative real-time PCR (RT-qPCR) using a QuantStudio™ 5 (Thermo Fisher Scientific Inc., Foster City, CA, USA) with Fast SYBR™ Green Master Mix (Applied Biosystems, Thermo Fisher Scientific Inc., Foster City, CA, USA). GAPDH was used as the endogenous control gene. The primer sequences used for amplification are listed in Table 3.

The thermal cycling protocol consisted of an initial denaturation step at 95 °C for 10 min, followed by 40 cycles of 95 °C for 15 s, 60 °C for 1 min, and a final dissociation stage of 95 °C for 15 s and 60 °C for 1 min. Each sample was analyzed in triplicate. Cycle threshold (Ct) values were determined using Design and Analysis Software 2.8.0 (Thermo Fisher Scientific Inc., Foster City, CA, USA). Relative gene expression was calculated using the 2^−ΔΔCt^ method [51], with GAPDH as the housekeeping gene and the differentiated control (C) as the reference condition.

### 4.9. Statistical Analysis

All experiments were performed in triplicate (three independent experiments), each with six technical replicates. Results from MTT and Oil Red O assays were expressed as a percentage relative to the differentiation control (C). Data were analyzed using one-way ANOVA, followed by Šidák’s multiple comparisons test for pairwise comparisons between groups, as these data met the assumptions of normality (assessed by the Shapiro–Wilk test) and homogeneity of variances. For prostanoid analysis, which did not meet these assumptions, pairwise comparisons were performed using the Mann–Whitney U test.

For RT-qPCR data, comparisons between multiple groups were performed using one-way ANOVA, followed by Dunnett’s post-hoc test for comparisons with the differentiation control (C), for each gene.

Results are presented as the mean ± standard error of the mean (SEM). Results were considered significant at *p* < 0.05. All analyses were performed using GraphPad Prism (version 8.0.2).

## 5. Conclusions

In conclusion, our findings demonstrate that *Vitis vinifera* and *Allium sativum* extracts inhibit adipogenesis in 3T3-L1 cells through distinct modulation of the COX pathway. The grape seed extract (GE), rich in OPCs, showed superior anti-adipogenic efficacy. At the prostanoid level, it promotes an anti-adipogenic prostanoid profile characterized by increased PGE_2_ and decreased TXA_2_, despite an increase in PGI_2_, suggesting that its transcriptional effects override any potential pro-adipogenic signal from PGI_2_. At the molecular level, GE exerts its potent effect primarily through transcriptional suppression of the master regulators PPARγ and CEBPα, accompanied by near-complete inhibition of the adipocyte maturation marker GLUT4, surpassing the effect of genistein, a known inhibitor of adipogenesis. In contrast, the garlic extract (ALL), rich in thiosulfinates, increases PGE_2_ while decreasing both TXA_2_ and PGI_2_. However, ALL does not suppress PPARγ, CEBPα, or GLUT4 at the transcriptional level, indicating a post-transcriptional mechanism of action. This distinct prostanoid profile, combined with the lack of transcriptional inhibition, suggests that ALL’s anti-adipogenic effect is mediated primarily through prostanoid receptor signaling rather than direct transcriptional suppression of adipogenic master regulators. The combination of both extracts (AGE) did not enhance the anti-adipogenic effect; instead, it was less effective than GE alone, indicating functional antagonism. The lack of synergy underscores the complexity of combining bioactive compounds from different natural sources and highlights that combinations do not always yield synergistic benefits and may even lead to functional antagonism. These results contribute to the growing body of evidence supporting the potential use of natural extracts as functional ingredients for obesity management and open new avenues for investigating the mechanistic basis of their anti-adipogenic properties. Our integrated phenotypic and molecular data provide a foundation for future studies aimed at elucidating the specific molecular targets of these extracts in adipocytes, including the analysis of post-transcriptional mechanisms, the role of prostanoid receptors, and the temporal dynamics of prostanoid biosynthesis during adipogenesis.

## Figures and Tables

**Table 1:** Phytochemical composition of the grape seed extract (GE), garlic extract (ALL), and their combination (AGE) at 600-fold dilution, as determined by HPLC.

Compound	GE (ppm)	ALL (ppm)	AGE 600 × Diluted (ppm)
Gallic acid	2658	—	0.22
Proanthocyanidin B1	10,260	—	0.85
Proanthocyanidin B2	23,348	—	1.95
Proanthocyanidin C1	7752	—	0.65
Epicatechin	34,091	—	2.84
Epigallocatechin	10,192	—	0.85
Oligomeric Proanthocyanidins (OPCs)	902,491	—	75.2
Dimethyl thiosulfinate	—	18,302	0.70
Allyl-methyl + methyl-allyl thiosulfinate	—	4581	0.18
Propyl-methyl + methyl-propyl thiosulfinate	—	3394	0.13
Diallyl thiosulfinate (Allicin)	—	5617	0.22
Allyl-1-propenyl thiosulfinate	—	31,023	1.19
1-Propenyl-allyl + allyl-propyl thiosulfinate	—	1760	0.07
Propyl-allyl thiosulfinate	—	1591	0.06
Dipropyl thiosulfinate	—	1653	0.06

**Table 2:** Gradient program for HPLC analysis of grape seed extract.

Time (min)	Water (%)	Methanol (%)	Acetonitrile (%)	Formic Acid (%)
0	89	5.3	5.2	0.5
5	74	23.3	2.3	0.4
15	62	34.9	2.5	0.6
28 to 50	53	25.5	16.3	5.2

**Table 3:** Primer sequences used for amplification.

Protein	Gene	GeneID	Forward	Reverse
PPARγ	*Pparg*	19016	5′CCATTGAGTGCCGAGTCTGT3′	5′AGGCACTTCTGAAACCGACA3′
C/EBPα	*Cebpa*	12606	5′CCGATGAGCAGTCACCTCC3′	5′AGGAACTCGTCGTTGAAGGC3′
GLUT4	*Slc2a4*	20528	5′CGGCTCTGACGATGGGGAA3′	5′CCACGTTGCATTGTAGCTCTG3′

PPARγ, peroxisome proliferator-activated receptor gamma; C/EBPα, CCAAT/enhancer-binding protein alpha; GLUT4, glucose transporter type 4.

## Data Availability

The original contributions presented in this study are included in the article/Supplementary Material. Further inquiries can be directed to the corresponding author.

## Supplementary Materials

The following supporting information can be downloaded at: https://www.mdpi.com/article/10.3390/ijms27157043/s1. References [52,53] are cited in Supplementary Materials.

## Abbreviations

The following abbreviations are used in this manuscript:

OPCs	Oligomeric proanthocyanidins condensed
GE	*Vitis vinifera* extract
ALL	*Allium sativum* extract
AGE	Combination *Vitis vinifera* and *Allium sativum* extracts
