# Supplementary material for: Effect of Combined Grape Seed (Vitis vinifera) and Garlic (Allium sativum) Extracts on Adipogenesis in the 3T3-L1 Preadipocyte Line
Source: Int J Mol Sci. 2026 Aug 6;27(15):7043. doi: 10.3390/ijms27157043 (PMC13467438; doi:10.3390/ijms27157043)
Supplement: Supplementary file 1 [file ijms-27-07043-s001.zip › ijms-4243458-supplementary.pdf]

Supplementary material

Effect of combined extracts of Grape Seed (Vitis vinifera) and Garlic (Allium sativum) on adipogenesis in the 3T3-L1 preadipocyte line

Supplementary Material S1: Dose-response analysis of Vitis vinifera and Allium sativum extracts in 3T3-L1 cells

Experimental design

To select the optimal concentration for subsequent experiments, 3T3-L1 preadipocytes were treated with serial dilutions (1:500, 1:600, 1:700, and 1:800) of grape seed extract (GE), garlic extract (ALL), and their combination (AGE) throughout the differentiation protocol. Cell viability was assessed by MTT assay at the end of differentiation and adipogenesis was evaluated by Oil Red O (ORO) staining to quantify intracellular lipid accumulation. All experiments were performed in triplicate with six technical replicates each.

Results

To assess the potential cytotoxic effects of the extracts, cell viability was evaluated by MTT assay on day 10 of differentiation. As shown in **Supplementary Figure S1, Panel A**, none of the tested dilutions of GE, ALL, or AGE significantly reduced cell viability compared to the control (C). Similarly, **Supplementary Table S1** reports the mean viability values, which remained above 93% for all conditions, confirming that the extracts were non-cytotoxic across the entire concentration range tested.

The effects of the extracts on adipogenesis were evaluated by Oil Red O staining to quantify intracellular lipid accumulation (**Supplementary Figure S1, Panel B**). Except for ALL at 1:800 dilution, all treatments significantly reduced lipid accumulation compared to control, with the most pronounced effects observed at the 1:500 and 1:600 dilutions. These findings are detailed in **Supplementary Table S1**, which presents the ORO values as mean percentage relative to control. The anti-adipogenic effect was dose-dependent for GE and AGE, whereas ALL showed significant inhibition only at dilutions 1/600 and lower.

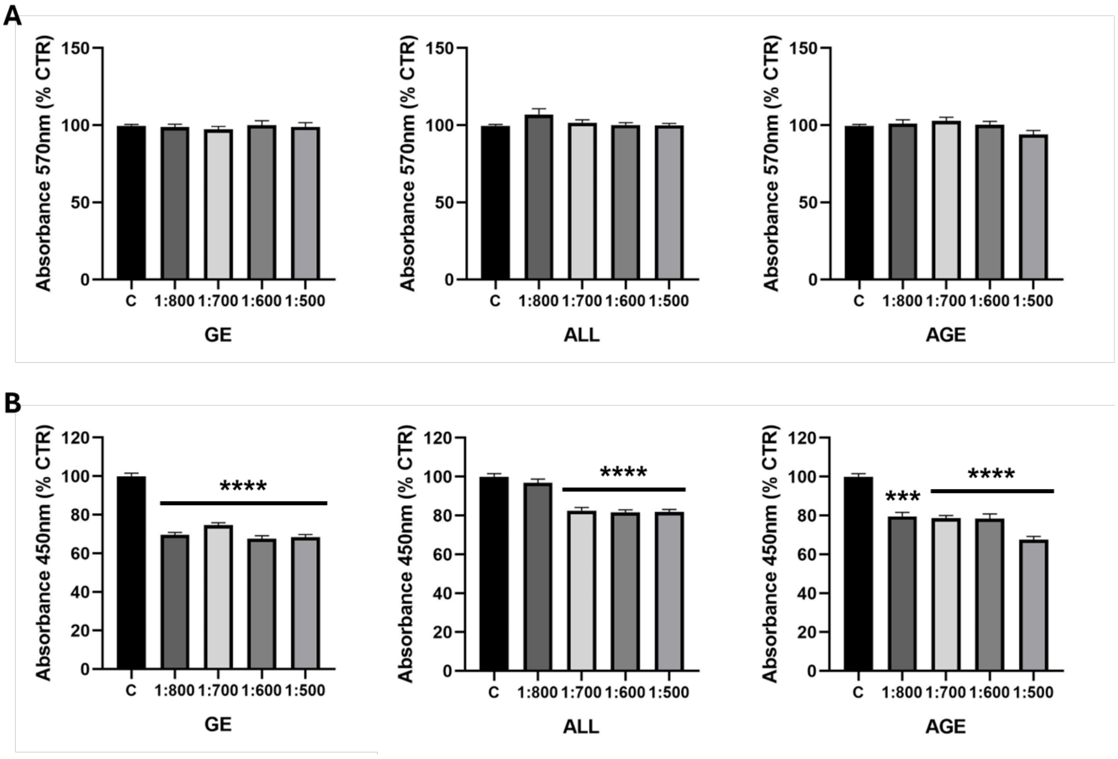

Supplementary Figure S1. Dose-response analysis of grape (GE), garlic (ALL), and combined (AGE) extracts in 3T3-L1 cells. Differentiating preadipocytes were treated with serial dilutions (1:500 to 1:800) throughout the differentiation protocol. (A) Cell viability assessed by MTT assay at day 10. (B) Intracellular lipid accumulation assessed by Oil Red O (ORO) staining at day 10. Data are presented as mean percentage relative to control (C) ± SEM (three independent experiments). ***p < 0.001, ****p < 0.0001 vs. control (one-way ANOVA with Dunnett's multiple comparisons test).

Supplementary Table S1. Effect of increasing dilutions of GE, ALL, and AGE on cell viability and lipid accumulation in 3T3-L1 cells.

Dilution	Parameter	GE (n)	ALL (n)	AGE (n)
**1/500**	MTT (% of C)	98.9 ± 11.5 (18)	99.1 ± 9.2 (54)	93.9 ± 11.1 (17)
	ORO (% of C)	68.0 ± 4.9**** (13)	81.8 ± 9.0**** (48)	68.0 ± 6.4**** (16)
**1/600**	MTT (% of C)	100.0 ± 13.0 (20)	100.1 ± 9.0 (37)	100.3 ± 10.9 (24)
	ORO (% of C)	68.0 ± 6.3**** (16)	81.6 ± 8.8**** (44)	78.0 ±9.8**** (17)
**1/700**	MTT (% of C)	97.2 ± 9.6 (24)	101.6 ± 6.9 (13)	102.8 ± 11.3 (23)
	ORO (% of C)	75.0 ± 4.2**** (13)	82.6 ± 6.6** (17)	79.0 ± 5.6**** (16)

**1/800**	MTT (% of C)	98.7 ± 9.8 (22)	104.5 ± 8.2 (7)	101.2 ± 11.8 (22)
	ORO (% of C)	70.0 ± 4.7**** (16)	96.8 ± 7.2 (14)	80.0 ± 7.9*** (15)

Data are presented as mean ± SEM (n). Significance levels vs. control: **p < 0.01, ***p < 0.001, ****p < 0.0001 (one-way ANOVA with Dunnett's multiple comparisons test).

Supplementary Material S2: Lipid accumulation normalized by cell viability (ORO/MTT ratio)

Rationale

To ensure that the observed differences in lipid accumulation were not confounded by subtle variations in cell viability, the Oil Red O (ORO) absorbance values were normalized by MTT absorbance values for each experimental condition. This ratio (ORO/MTT) provides a more accurate measure of adipogenesis per viable cell. Data are presented as percentage relative to the positive differentiation control (C).

Calculations

For each condition, the ORO/MTT ratio was calculated as:

$$\text{ORO/MTT ratio}=\frac{\text{Mean ORO absorbance}}{\text{Mean MTT absorbance}}$$

The ratio was then expressed as a percentage of the control (C):

$$\text{Normalized ratio (\% of C)}=\left(\frac{\text{ORO/MTT ratio\_condition}}{\text{ORO/MTT ratio\_C}}\right)\times 100$$

Standard errors for the ratios were calculated using the formula for propagation of errors in division:

$$\text{SEM\_ratio}=\text{ORO/MTT ratio}\times\sqrt{\left[\left({}^{\text{SEM\_ORO}}/_{\text{Mean\_ORO}}\right)^2+\left({}^{\text{SEM\_MTT}}/_{\text{Mean\_MTT}}\right)^2\right]}$$

Where:

$\text{SEM\_ORO}={}^{\text{SD ORO}}/_{\sqrt{(n\_ORO)}}$

$\text{SEM\_MTT}={}^{\text{SD MTT}}/_{\sqrt{(n\_MTT)}}$

Statistical analysis

Data were analyzed using one-way ANOVA followed by Tukey's multiple comparisons test for pairwise comparisons between groups. For comparisons

involving control (C), Dunnett's test was applied. Statistical significance was set at $p < 0.05$.

Results

To account for any potential confounding effects of cell viability on lipid accumulation, ORO values were normalized by MTT values for each condition. The resulting ORO/MTT ratios, expressed as a percentage of the positive control (C), are presented in **Supplementary Table S2** and graphically depicted in **Supplementary Figure S2**.

The non-differentiated negative control (CTR-) showed significantly lower ORO/MTT ratios compared to positive control of differentiation (C) across all conditions, confirming the validity of the differentiation model. All three extracts significantly reduced the ORO/MTT ratio compared to C for all dilutions, confirming that the anti-adipogenic effects are not attributable to cytotoxicity.

GE exhibited the most potent anti-adipogenic effect at the selected working dilution (1/600), with an ORO/MTT ratio of 68.4 ± 3.4% of control. This was significantly lower than both ALL (77.5 ± 3.3%, $p < 0.0001$) and AGE (73.6 ± 4.0%, $p < 0.01$) as shown in **Supplementary Table S3**. In contrast, ALL and AGE showed similar effects at 1/600 dilution, with no statistically significant difference between them. The combination treatment (AGE) did not enhance the anti-adipogenic effect compared to GE alone at any dilution, indicating that the combination does not produce synergistic benefits.

Regarding the dose-response relationship, dose-dependent trends were observed for all extracts, with the lowest ORO/MTT ratios generally achieved at the highest concentrations (1/600 and 1/500). While minor fluctuations were observed at intermediate dilutions, the overall pattern supports a dose-dependent anti-adipogenic effect

Supplementary Table S2. ORO/MTT ratio normalized to positive control (C) for all treatments and dilutions. Results are presented as mean ± SEM.

**Extract**	**Dilution**	**ORO/MTT ratio (% of C)**	**SEM**	**n (ORO/MTT)**	**Significance vs. C**
**GE**	CTR-	80.8	4.2	7/9	****
	1/800	71.3	3.2	16/22	****

	1/700	76.0	3.0	13/24	****
	1/600	68.4	3.4	16/20	****
	1/500	67.5	3.5	13/18	****
**ALL**	CTR-	93.5	3.9	9/2	ns
	1/800	84.7	3.6	14/7	**
	1/700	77.1	3.4	17/13	****
	1/600	77.5	3.3	44/37	****
	1/500	76.2	3.2	48/54	****
**AGE**	CTR-	82.5	4.6	7/13	***
	1/800	74.2	3.9	15/22	****
	1/700	72.3	3.7	16/23	****
	1/600	73.6	4.0	17/24	****
	1/500	66.9	3.8	16/17	****

Negative control (CTR-) represents non differentiated cells. Significance levels vs. C: ns = not significant; **p < 0.01; ***p < 0.001; ****p < 0.0001*

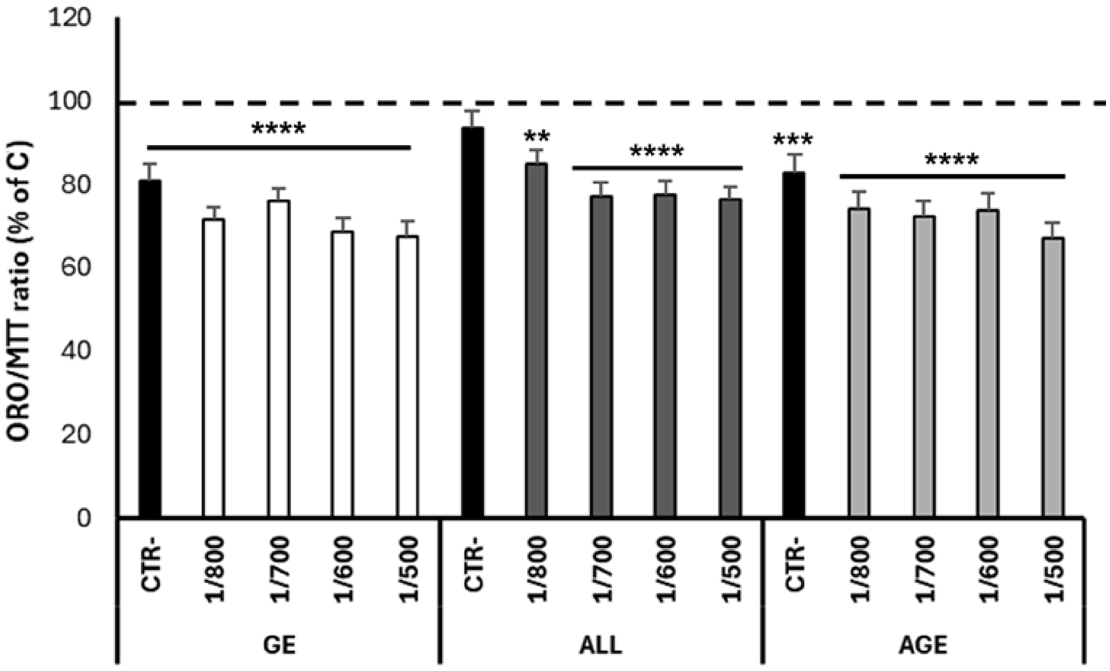

Supplementary Figure S2. Dose-response curves for ORO/MTT ratio (lipid accumulation normalized by cell viability) in 3T3-L1 cells treated with dilutions of GE, ALL, and AGE. Negative control (CTR-) represents non differentiated cells. Data are presented as mean ± SEM. The horizontal dashed line represents the control (C = 100%). Statistical significance vs. C: *p < 0.05, **p < 0.01, ***p < 0.001, ****p < 0.0001 (one-way ANOVA with Dunnett's test).

Supplementary Table S3. Pairwise statistical comparisons between treatment groups at the selected working dilution (1/600) for ORO/MTT ratio.

**Comparison**	**GE (1/600)**	**ALL (1/600)**	**AGE (1/600)**

**GE (1/600)**	-	****	**
**ALL (1/600)**	****	-	ns
**AGE (1/600)**	**	Ns	-

Significance levels: ns = not significant; **p < 0.01; ****p < 0.0001 (Tukey's multiple comparisons test).

Selection of working concentration based on normalized data

The normalized ORO/MTT ratio data confirm the appropriateness of selecting the 1/600 dilution for subsequent experiments: At 1/600 dilution, all extracts maintained significant anti-adipogenic effects (p < 0.0001 vs. C for all treatments); GE showed maximal effect at 1/600 (68.4%), with no further reduction at 1/500 (67.5%, ns); ALL and AGE showed similar effects at 1/600 and 1/500, indicating that the anti-adipogenic effect plateaus at this concentration range; The normalization by MTT confirms that the effects are not confounded by variations in cell viability.

Selection of working concentration

In summary, based on these results, the 1:600 dilution was selected for all subsequent experiments based on the following criteria: First, it maintained cell viability above 95% for all treatments, confirming the absence of cytotoxicity; second, it produced significant inhibition of adipogenesis across all treatments (p < 0.001 for GE and AGE; p < 0.01 for ALL); third, it represented the lowest concentration (highest dilution) that sustained maximal anti-adipogenic activity, providing a conservative estimate of extract bioactivity and fourth, it corresponds to approximately 23 μg/mL allicin (for ALL) and 0.05% OPCs (for GE), concentrations that are physiologically relevant and achievable through dietary supplementation [52,53].

References for supplementary material

52. Salehi, B.; Zucca, P.; Orhan, I.E.; Azzini, E.; Adetunji, C.O.; Mohammed, S.A.; Banerjee, S.K.; Sharopov, F.; Rigano, D.; Sharifi-Rad, J.; et al. Allicin and health: A comprehensive review. Trends in Food Science & Technology 2019, 86, 502-516, doi:https://doi.org/10.1016/j.tifs.2019.03.003.

53. Unusan, N. Proanthocyanidins in grape seeds: An updated review of their health benefits and potential uses in the food industry. Journal of Functional Foods 2020, 67, 103861, doi:https://doi.org/10.1016/j.jff.2020.103861.
